# Learning from a pitfall: Atrial septostomy in LV failure under VA-ECMO for pulmonary hypertension

**DOI:** 10.1051/ject/2026001

**Published:** 2026-06-19

**Authors:** Camille Wirth, Pierre De Normandie, Sebastien Hascoet, Elie Fadel, François Stephan

**Affiliations:** 1 Cardiothoracic Intensive Care Unit; Marie Lannelongue Hospital 133 Avenue de la Résistance 92350 Le Plessis Robinson France; 2 Department of Pediatric Cardiology, Catheterism and Rythmology, Marie Lannelongue Hospital 133 Avenue de la Résistance 92350 Le Plessis Robinson France; 3 University Paris-Saclay, Faculty of Medicine Kremlin-Bicêtre France; 4 INSERM U999, Pulmonary Hypertension: Pathophysiology and Novel Therapies, Marie Lannelongue Hospital Le Plessis-Robinson France; 5 Department of Thoracic and Vascular Surgery and Heart-Lung Transplantation, Marie Lannelongue Hospital 133 Avenue de la Résistance 92350 Le Plessis Robinson France

**Keywords:** Venous arterial extra corporeal oxygenation, Pulmonary hypertension, Stress-induced cardiomyopathy, Left ventricular failure, Atrio-septostomy, Case report

## Abstract

*Background*: Atrial septostomy has been successfully used in patients with pulmonary hypertension and in those supported with veno-arterial extracorporeal membrane oxygenation (VA-ECMO) complicated by pulmonary congestion. *Case description*: We report a patient on VA-ECMO for decompensated pulmonary hypertension who subsequently developed stress-induced cardiomyopathy and pulmonary congestion. An atrial septostomy was performed, which led to worsening respiratory status due to the creation of a right-to-left shunt, and hemodynamic deterioration resulting from increased left ventricular preload. *Conclusion*: The implications of atrial septostomy should be carefully considered prior to decompression, as there may be situations, especially if right-sided pressures are less than left-sided pressures, where patients may still benefit.

## Introduction

Among the recognized indications, atrial septostomy (AS) has been successfully performed in patients with pulmonary hypertension (PH) [1] and in patients with circulatory failure supported by veno-arterial extracorporeal membrane oxygenation (VA-ECMO) [2–4]. However, the goals of AS differ markedly between these two settings. AS in PH is to maintain left ventricular preload at the expense of oxygen saturation [1], and AS in VA-ECMO is to allow for left-to-right shunting to unload the left ventricle (LV) and mitigate left atrial (LA) hypertension [2].

We report the case of a patient who required VA-ECMO for decompensated PH and subsequently developed stress-induced cardiomyopathy with pulmonary congestion. AS was performed, leading to a deterioration of both respiratory and hemodynamic parameters. We discuss the mechanisms underlying this deterioration and suggest that AS should not be considered in this specific clinical context.

## Case presentation

A 38-year-old woman (gravity 1 and parity 1) with no significant comorbidities, except for treated hypothyroidism, was admitted to a regional hospital with circulatory shock due to decompensated pulmonary hypertension (pH 7.17, HCO_3_⁻ 14.4 mmol/L, lactate 10 mmol/L, PaO_2_ 29 mmHg). Transthoracic echocardiography revealed a dilated right atrium (RA) and right ventricle (RV) (tricuspid annular plane systolic excursion (TAPSE) 8 mm, S′ velocity 9 cm/s), and a D-shaped LV with preserved LV systolic function. NT-proBNP was elevated at 10.748 pg/mL. Computed tomography excluded acute pulmonary embolism. Right heart catheterization demonstrated a mean pulmonary arterial pressure of 58 mmHg, cardiac output of 1.5 L/min, pulmonary capillary wedge pressure of 7 mmHg, and RA pressure of 20 mmHg. She also presented with acute renal dysfunction (serum creatinine 192 μmol/L, serum urea 13.8 mmol/L) and hepatic failure (prothrombin activity 24%, AST 1575 U/L, ALT 900 U/L). The patient was initiated on VA-ECMO while maintaining spontaneous breathing with supplemental oxygen without respiratory assistance (height 155 cm, weight 70 kg, left femoral arterial cannula 19F, right femoral venous cannula 23F). Her clinical status improved dramatically under VA-ECMO, and two days later, she was transferred to our intensive care unit for consideration of bilateral lung transplantation.

A repeat echocardiogram confirmed persistent RV failure but revealed severely impaired LV contractility, with an ejection fraction estimated at 10–15%. A diagnosis of midventricular atypical stress cardiomyopathy was established [5] (start of the Q wave to the end of the T wave on electrocardiogram and corrected for heart rate (QTc) > 510 ms, normal coronary angiography, and mild elevation of troponin I at 1910 ng/L). On the third day after VA-ECMO initiation, the patient developed acute respiratory failure associated with systemic arterial hypertension (mean arterial pressure 120 mmHg). Pulmonary congestion gradually improved with blood pressure control and non-invasive ventilation.

**Figure 1:**
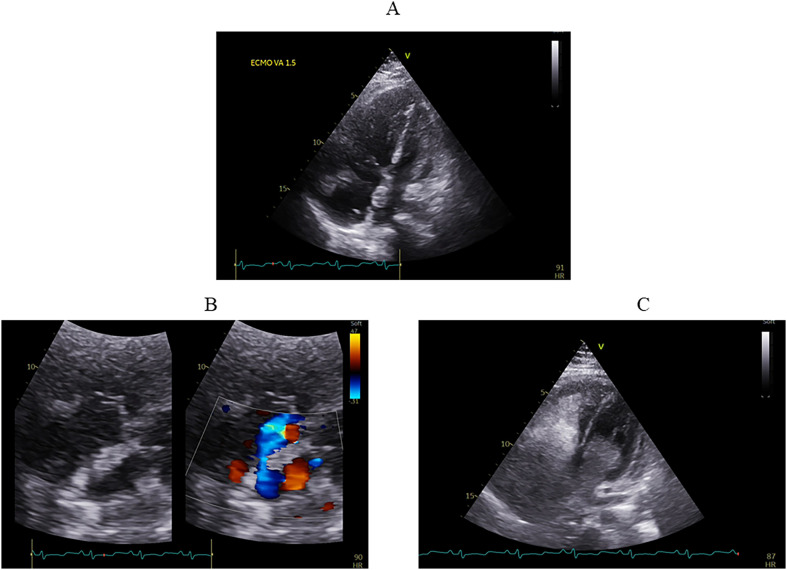
A. Apical four-chamber view on transthoracic echocardiography performed before atrioseptostomy (veno-arterial ECMO flow = 1.5 L/min). The view showed a dilated right ventricle, a compressed D-shape left ventricle, and a flattened septum. B. Transthoracic echocardiography with Color Doppler. A turbulent blood flow is visible through the interauricular septum. The flow appears bidirectional (with both blue and red signals mixed). The peak velocity is indicated around 47 cm/s, with a color scale ranging from −31 to +47 cm/s. C. Transthoracic echocardiography with a bubble contrast study. The bubble contrast study shows an opacification of the right-sided chambers and an immediate left heart opacification, suggesting an overt shunt.

With the aim of accelerating clinical improvement and preventing recurrence of pulmonary edema despite the introduction of medical treatment, AS was decided. Transthoracic echocardiography performed before atrioseptostomy (veno-arterial ECMO flow = 1.5 L/min) showed a dilated RV, a compressed D-shape LV, and a flattened septum (Figure 1A). Balloon AS was performed via a femoral venous approach, using transseptal puncture with a Brockenbrough needle followed by two static balloon dilations (30 mm). Immediately after the procedure, the patient’s respiratory status deteriorated, with hypoxemia, tachypnea, and decreased pulse pressure. She stabilized but remained hypoxemic. Serial echocardiographic parameters are summarized in Table 1.

**Table 1:** Course of hemodynamic and respiratory variables before and after atrioseptostomy procedure.

Variables	Day −1 atrioseptostomy	Day 0		Day +1 atrioseptostomy
		Before atrioseptostomy	After atrioseptostomy	
VA ECMO parameters				
Revolutions/min	3100	3035	3016	2924
Sweep gas flow (L/min)	1	1	1	1
FiO_2_ to the membrane lung	0.80	0.80	1	1
Extracorporeal blood flow (L/min)	3.45	3.54	3.51	3.36
PaO_2_ (mmHg)	118	94	57	75
PaCO_2_ (mmHg)	31	32	37	32
FiO_2_ to the native lung	0.21	0.21	0.60	0.60
Respiratory rate/min	21	26	36	25
Radiologic score*	0	4	7	8
Mean arterial pressure (mmHg)	86	90	83	82
Heart rate (beat/min)	92	102	84	88
Pulse pressure (mmHg)	20	24	12	22
Central venous pressure (mmHg)	–	19	–	–
Left atrial pressure (mmHg)	–	12	–	–
Echocardiographic parameters				
LVEF (%)	15	15	15	20
LV end-diastolic diameter (mm)	48	48	51	48
RV/LV ratio	1.17	1.04	0.80	0.72

VA-ECMO: venous arterial extra corporeal membrane oxygenation; LVEF: left ventricular ejection fraction; LV: left ventricular; RV: right ventricular.

* Radiologic score: the anterior–posterior chest roentgenograms were divided into four zones using a horizontal line originating from the hilus; each zone was then graded as follows: 0, normal; 1, interstitial pulmonary infiltrates; 2, fluffy alveolar infiltrates; 3, dense alveolar infiltrates. Thus, the score could range from 0 to 12, with higher scores indicating greater severity of infiltration.

A follow-up echocardiogram performed three days later demonstrated clear improvement in LV function (LV ejection fraction 45%) and confirmed a right-to-left shunt (Figures 1B and 1C). The patient subsequently underwent bilateral lung transplantation eight days later while she was still on VA-ECMO, with a favorable postoperative course.

## Discussion

**Figure 2:**
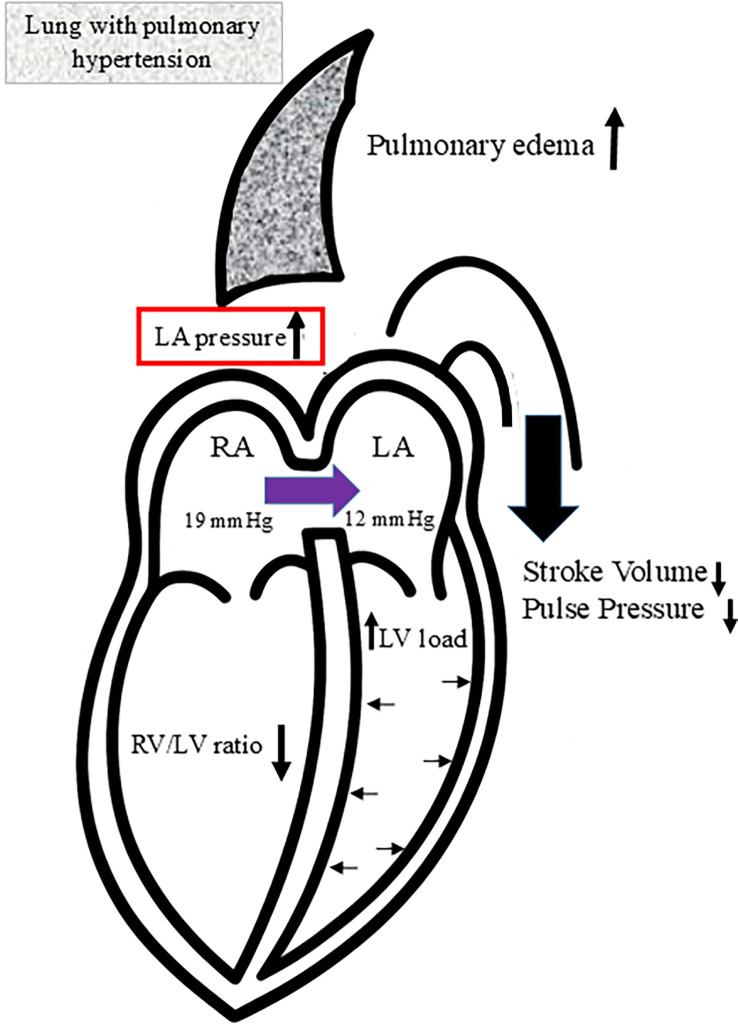
Effect of atrio-septostomy (purple arrow) in case of pulmonary hypertension and left ventricular failure in a patient under veno-arterial extra corporeal membrane oxygenation.

This case highlights the potential harmful effects of AS in the setting of LV failure associated with primary PH (Figure 2). In PH patients, AS has been reported to consistently induce a 7–10% decrease in systemic arterial oxygen saturation, followed by improved oxygen delivery and durable hemodynamic benefits at rest [1]. Peripheral VA-ECMO is the most frequently used support modality for RV failure in PH patients, despite the associated increase in LV afterload [6]. Because the LV is usually preserved in PH, the effect of VA-ECMO–induced afterload elevation is typically negligible.

In our case, however, stress-induced cardiomyopathy developed [1], resulting in severe LV dysfunction with pulmonary congestion. LV overload under VA-ECMO depends mainly on afterload pressure [7, 8], intrinsic LV contractility [8], and ECMO flow [7]. Peripheral vasodilators are generally effective in reducing afterload and improving respiratory status [7, 8]. When medical management fails, left heart decompression becomes mandatory. Several LV venting strategies have been described [3], and AS has proven effective in expert centers [2, 4, 9]. The primary objective of AS is to reduce LA pressure. Accordingly, AS should only be considered when echocardiography demonstrates left-to-right bulging of the interatrial septum or when direct LA pressure measurements confirm elevation [10]. In such conditions, AS can provide immediate and substantial LV unloading. Similarly, AS may be beneficial in LV failure associated with *passive* pulmonary hypertension [11].

In the present case, the absence of LA dilation and elevated LA pressure, combined with markedly increased RA pressure, likely accounted for the detrimental effect of AS. First, hypoxemia was aggravated by AS through the creation of a right-to-left shunt [1] (Table 1 and Figures 1B and 1C), in addition to the development of pulmonary edema, as demonstrated by the radiologic score. Second, the shunt likely contributed to worsening LV overload, despite only moderately elevated left-sided pressures before AS [1]. Although direct LA pressure was not measured after the procedure, a rise in pressure can be anticipated based on hemodynamic principles [1]. Such an acute increase in LV filling pressure likely resulted in higher ventricular wall tension and myocardial stretch, thereby worsening contractility [12] and leading to reduced stroke volume and pulse pressure (Table 1). Conversely, AS did achieve partial decompression of the right heart chambers, as reflected by a decrease in the RV/LV ratio (Table 1 and Figure 1C).

It remains uncertain whether left ventricular unloading was a requisite procedure for this patient. It is possible that systemic arterial pressure control would have been enough without the need for left ventricular unloading.

If the need for unloading persisted, there were other, more appropriate options available. The most frequently reported LV unloading intervention during veno-arterial extracorporeal membrane oxygenation (VA-ECMO) is the intra-aortic balloon pump (IABP) [10]. However, the unloading effect is rather limited, with a LA pressure decrease of around 5 mmHg [10]. In our patient, the IABP would have been sufficient in terms of the hemodynamic parameters. The use of the IMPELLA system was also an elegant solution, apart from its cost. Decrease of LA pressure unloading is expected to range between 4 mmHg and 17 mmHg [10]. The use of left atrial VA-ECMO (LAVA-ECMO) using a fenestrated cannula to drain the left and right atria provides active biventricular unloading [13]. This could also have been a solution to consider. The other alternatives do not seem appropriate, particularly those involving surgery [10].

In conclusion, the implications of atrial septostomy should be carefully considered prior to decompression, as there may be situations, especially if right-sided pressures are less than left-sided pressures, where patients may still benefit. Alternative strategies for LV venting are available and should be prioritized [3].

## Data Availability

The data that support the findings of this study are available from the corresponding author upon reasonable request.
